# Teaching protein structure and function through molecular visualization

**DOI:** 10.1002/bmb.21860

**Published:** 2024-09-04

**Authors:** Elisabeth Baland, Lucía Pérez Jimenez, André Mateus

**Affiliations:** ^1^ Department of Chemistry Umeå University Umeå Sweden; ^2^ Department of Molecular Biology Umeå University Umeå Sweden; ^3^ The Laboratory for Molecular Infection Medicine Sweden (MIMS) Umeå Sweden

**Keywords:** biochemistry, molecular graphics, motifs, protein structure and function, tutorials

## Abstract

The function of proteins is governed by their three‐dimensional structure. This structure is determined by the chemical characteristics and atomic interactions of amino acids. Students of biochemistry, with a particular focus on protein chemistry, benefit from looking at protein structures and understanding how proteins are built and fold. Due to their three‐dimensional nature, static two‐dimensional representations in textbooks can be limiting to student learning. Here, we developed a series of tutorials that introduce students to molecular graphics software. The students are challenged to apply the software to look at proteins and to get a deeper understanding of how amino acid properties are linked to structure. We also familiarize students with some of the latest tools in computational structural biology. Students performed the tutorials with visual enthusiasm and reported general satisfaction in being able to visualize theoretical concepts learned during lectures. We further stimulated student engagement by allowing space for self‐exploration. We share the tutorial instructions for other teachers to build on them, and we also offer suggestions for further improvement based on student feedback. In summary, we present a series of tutorials aimed at students of an advanced course in protein biochemistry to enable them to explore the universe of protein structures and how those relate to function.

## 1 INTRODUCTION

The three‐dimensional (3D) structure of proteins defines their function. This 3D structure is determined by the physicochemical characteristics of the sequence of amino acids and interactions between their atoms. The structure of a protein can be experimentally resolved by using methods such as X‐ray crystallography, nuclear magnetic resonance (NMR), or cryogenic electron microscopy (cryoEM). Over 200,000 protein structures have been deposited in the protein data bank (PDB).^1^ These data have been used to train deep learning methods to predict protein structures,^2^, ^3^, ^4^ which has resulted in virtually all known protein sequences now having a predicted structure.^5^

Most students of biochemistry encounter protein structures only as static two‐dimensional illustrations in textbooks. However, there are multiple tools available for visualizing protein structures.^6^ One example is ChimeraX,^7^ a free software for non‐commercial use. The development team has compiled multiple tutorials for specific tasks,^8^ but it can be challenging for a student to know where to start.

For that reason, as part of an advanced course in protein chemistry, we have developed a series of five teaching moments focused on molecular graphics. These consist of structured step‐by‐step tutorials that introduce students to molecular graphics software, and lead them to explore the physicochemical characteristics of proteins, the interactions between their atoms, motifs and domains, and how they can use other computational tools to get hints on protein function. By allowing students to manipulate 3D representations of protein structures, we aimed at deepening the students understanding of protein chemistry,^9^, ^10^, ^11^, ^12^ and prepare them to use the latest tools in computational structural biology.

## 2 MATERIALS AND METHODS

### 2.1 Course structure

The teaching moments described in this manuscript are part of the course “Biochemistry: protein structure and function course” at Umeå University in Sweden. This is a 2.5‐month course given at the master level and requiring students to have at least a 2.5‐month education in basic biochemistry. The syllabus can be found in Data S1 and the intended learning outcomes (ILOs) are that by the end of the course the students should be able to:

- Identify different motifs and domains in protein structures and how these relate to function.
- Understand and use the thermodynamic principles that determine the structure and folding of proteins, how and these impact function.
- Understand and describe the methodologies to determine protein structure, and suggest which one is more adequate to study a particular protein.
- Use the principles of molecular recognition to understand interactions with proteins.
- Relate the theoretical framework of catalysis to its application to study enzyme function.
- Use computational tools to visualize protein structure and to predict their function.
- Design and perform experiments to study which aspects of the structure are important for the stability, structure, and function of proteins.
- Critically discuss the results of experiments designed to study protein stability, structure, and function.

**FIGURE 1:**
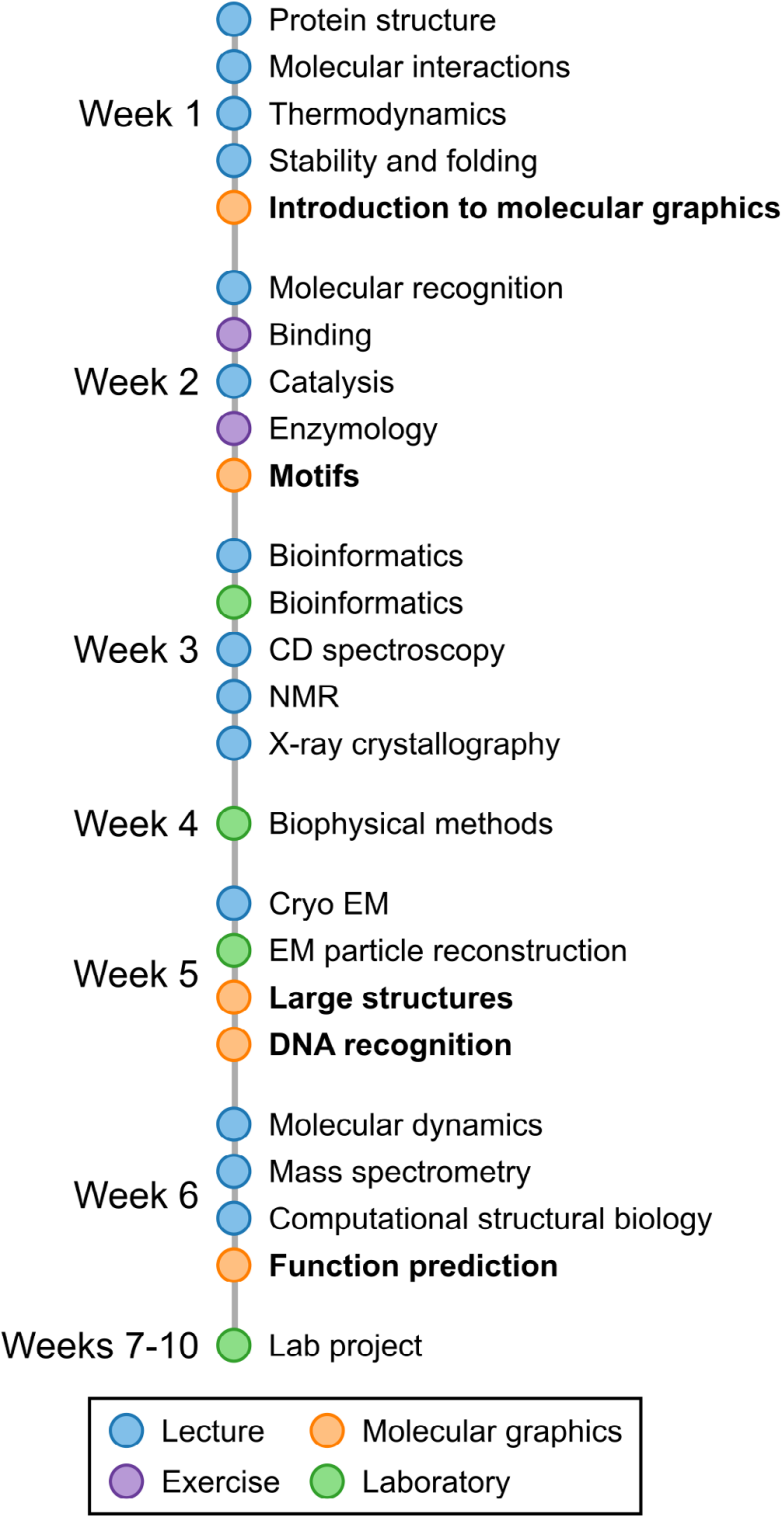
Timeline for the course “Biochemistry: protein structure and function course” at Umeå University. The course comprises lectures and laboratory moments on different aspects of protein chemistry and methodologies to determine protein structure, interactions, and function. To help students visualize protein structures, we have designed a series of five hands‐on tutorials with molecular graphics software (highlighted in the figure).

To achieve the ILOs, this course comprises a series of lectures on basic principles of physicochemical properties of proteins, protein stability and folding, protein interactions and catalysis, and introduction to different methods to determine protein structure, interactions, and function (Figure 1). These concepts are applied during laboratory moments on bioinformatics, biophysical methods, and particle reconstruction from cryoEM data, as well as a final project. Throughout the course, the students participate in a series of five hands‐on step‐by‐step tutorials using the molecular graphics software ChimeraX,^7^ and are encouraged to use the software in other teaching moments.

### 2.2 Designing the molecular graphics moments

We designed tutorials with the major goal of introducing students to molecular graphics software and allowing them to directly interact with protein structures. The tutorials can be found in Data S2. These incorporated aspects of previously published approaches,^10^, ^11^, ^12^ and of an older version of these tutorials. That version had received some criticism from the students during the course evaluations. For example, one of the students reported “The molecular graphics class could be more structured […] it was easy to feel lost, especially at the beginning,” while another student suggested “Change the exercises so that they bring something else than just look at a protein in 3D […] The purpose of those sessions was not clear”, and another student offered “The molecular graphics part could be improved, sometimes it was really confusing what we had to do.”

With this in mind, we decided to structure our tutorials in five sessions spread throughout the course and linked to the rest of the curriculum (Figure 1). The sessions also included recently developed computational tools that are now routinely used in structural biology.^2^, ^13^, ^14^ The first four sessions were scheduled for 2 h each, and the last session was scheduled for 4 h. The topics of each of the sessions were:

1. Introduction to molecular graphics.
2. Motifs in protein structures.
3. Larger structures.
4. DNA recognition.
5. Getting hints on protein function using computational tools.

**FIGURE 2:**
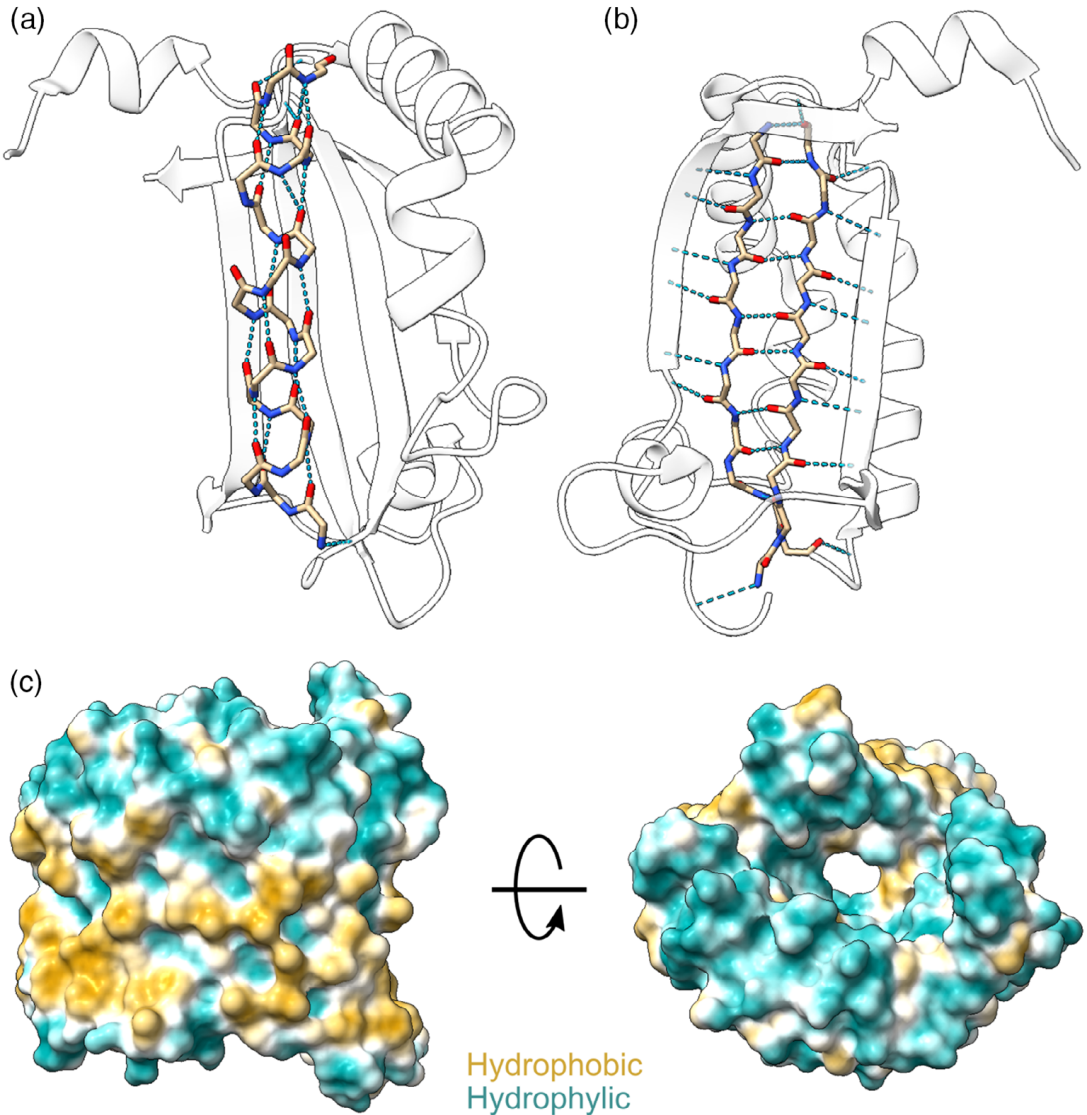
Physicochemical properties and interactions of amino acids determine protein structure. Hydrogen bonds are essential for providing stability to (a) alpha helices and (b) beta sheets (PDB: 2OKQ). (c) Hydrophobic side chains promote the insertion of transmembrane proteins in the membrane, while the hydrophilic interior allows the passage of polar molecules (PDB: 1A0S).

During session 1, the students learn to open PDB^1^ or AlphaFold models^2^ and get familiar with using the mouse actions, menus, and the command line. However, we attempted to have exercises that required minimum interaction with the command line to not overwhelm students that are not familiar with programming. The students also explore different representations of proteins (atoms/cartoons/surfaces), how to specifically color different aspects of the protein or how to measure distances. Finally, the students are trained to compare structures and to visualize possible movements in proteins. Students are asked to put these skills to practice by comparing the quality of AlphaFold models in relation to experimentally determined structures, and by visualizing atomic interactions in secondary structures of proteins, for example, how hydrogen bonding stabilizes alpha helices (Figure 2a) and beta sheets (Figure 2b).

During session 2, the students connect how the side chains of the different amino acids contribute to interactions (e.g., electrostatic interactions) and structure (e.g., the rigid structure of proline introduces kinks in alpha helices). The students also explore simple structural motifs such as the EF‐hand, hairpins or the Greek key, and how sequence motifs connect to structure.

During session 3, the students engage with larger structures, such as membrane proteins, and link the physicochemical properties of amino acids with their location on the structure, for example, hydrophobic side chains promote membrane insertion in transmembrane regions of proteins, while the hydrophilic interior contributes to the passage of polar solutes (Figure 2c).

During session 4, the students look at how proteins interact with nucleic acids, and how DNA can acquire different conformations.

During session 5, groups with approximately four students each are given a UniProt entry^15^ of an uncharacterized protein, and use traditional tools to identify functional motifs (such as BLAST^16^ or InterPro^17^), and recently developed tools to compare protein structures to the whole protein universe (e.g., Foldseek^13^) or to predict protein function (e.g., DeepFRI^14^). Their goal is to create the first steps to study the function of proteins. At the end of the session, the students share their strategies and findings with the rest of the class in the form of a short oral presentation.

### 2.3 Course evaluation

During the first implementation of these teaching moments, the students were asked to rank the sessions during the final course evaluation with the following prompt “This year, the molecular graphics sessions were greatly redesigned. How much did you like the format of the molecular graphics sessions? Tell your opinion if something could be improved.” They were given the chance to answer: “Not at all,” “Bad,” “OK,” “Good,” or “Very good,” as well as, input any free text in an online platform. All information was collected anonymously, and no personal data was collected that could identify the students, in accordance to the Swedish Ethical Review Act (2003:460).

## 3 RESULTS

We designed a series of tutorials to introduce students to molecular graphics software and to give them the opportunity to use it to explore aspects of protein chemistry. Further, the tutorials introduced students to recently developed computational tools that are now used routinely by structural biologists to understand protein function from structure.

The sessions were attended by all 17 students registered for the course. The students followed the tutorials individually, but were encouraged to ask their classmates how certain tasks were performed. To activate the students, the instructions omitted how certain tasks should be performed and instead pushed the students to look for solutions on their own, either by trial‐and‐error or by using a search engine or a large language model. The role of the teachers was to support students when they could not complete one of the assignments and to summarize the key concepts learned in each session. While the sessions were implemented in a small student cohort, their nature makes them scalable to a large number of students.

The sessions were generally well received by the students. The teachers noticed engagement from most students during the tutorials. The students generally communicated how visualizing aspects of protein structure in 3D helped them in understanding concepts they had learned during the lectures. The last session also provided an opportunity for students to experience the role of a researcher in structural biology. Although challenging, since for most proteins the students could not obtain much information, the students appreciated this moment.

**FIGURE 3:**
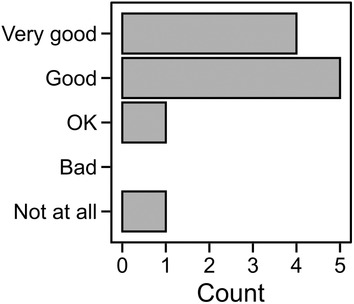
Answers from the students to “How much did you like the format of the molecular graphics sessions?” *n* = 11 students.

During the course evaluation, 11 students (65%) answered the question on what they thought of the sessions (Figure 3). The vast majority answered that they thought they were “Good” (45%) or “Very good” (36%). One student selected the lowest score, but unfortunately only commented “I didn't find it very useful.”, which does not provide a chance for improvement. The student that thought the sessions were “OK” reported “I think they were very clear, but sometimes in one session there was a lot of repetition and I think it was pretty clear to everyone, so this wasn't really necessary. […] However, it was very clear! I worked with it a lot before, but this was the first time it was actually explained.” Related to this, one of the other students wrote “It was a good exercise, but some sessions can be finished in one hour” (“Good”). Encouragingly, the other students stated that this was a “Great intro to molecular graphics! Maybe more “real‐life” examples, what this tool is used for in research” (“Very good”), “I think the way the molecular graphics sessions were made, made it easy to understand the lecture and helped to have a visual representation” (“Good”), “I really liked it, it was fun to identify and know more about the protein using different tools” (“Very good”), and “It was nice to experiment on the computer for the future” (“Good”).

In general, the students thought that these sessions were useful and increased their knowledge of how proteins obtain their structures.

## 4 DISCUSSION

Protein structure visualization is a common aspect in advanced courses in protein chemistry.^10^, ^11^, ^18^ In this work, we developed a new series of tutorials taking the students from the basic aspects of using molecular graphics software to applying it to understand how the structure of proteins is related to amino acid composition. We also introduced the students to novel computational tools that are now part of the toolkit of structural biology. We share these tutorials (Data S2) to inspire other teachers to build on them.

Despite providing step‐by‐step instructions on how to perform specific tests, we included some steps in which the students are stimulated to look on their own how to perform certain tasks, either by using a search engine or a large language model, such as chatGPT. This not only ensures that the tutorial stays relevant as novel computational structural biology tools become available and the students can find their way to such tools on their own, but it also promotes student engagement.^19^

Based on the student feedback, it is possible to shorten some of the sessions by reducing some steps that appear redundant. Alternatively, it is possible to include extra assignments for students that grasp concepts faster and want to further explore the capabilities of the molecular graphics software. One option is to make it clearer that students can explore the tutorials created by the developers of the software^8^—although this is already explicitly written in the instructions.

Another aspect that could be included is for students to predict their own protein structures by using cloud‐based services, such as ColabFold^20^ or AlphaFold server.^4^ Even though structures are available for all protein sequences present on UniProt,^5^ scientists routinely encounter novel sequences for which it is useful to predict a structure^21^ or want to model multimeric protein complexes. We are also considering including an extra session in which students would fit a molecular model to an electron microscopy map. This would be linked to the laboratory moment in which students perform particle reconstruction from cryoEM data following a tutorial created by the developers of CryoSPARC.^22^, ^23^ Finally, since protein structures are inherently three‐dimensional, and virtual/augmented reality becomes more accessible, some of the exercises could be performed in such an environment.^24^

## 5 CONCLUSION

In conclusion, visualizing protein structures can help students better understand why proteins fold into specific conformations and how some of those are linked to function. As part of an advanced course in protein chemistry, the series of tutorials presented here functions not only as an introduction to molecular graphics, but also in preparing the students to be able to use computational tools that are frequently used by structural biologists.

## AUTHOR CONTRIBUTIONS

All authors participated in the design of the teaching moment, performed the analysis, and wrote the manuscript.

## CONFLICT OF INTEREST STATEMENT

The authors declare no conflict of interest.

## Supporting information


**Data S1.** Syllabus and intended learning outcomes for “Biochemistry: protein structure and function course” at Umeå University in Sweden.

**Data S2.** Tutorials for the molecular graphics sessions.

## Data Availability

Data available in article Supporting Information.
