# Supplementary material for: Teaching protein structure and function through molecular visualization
Source: Biochem Mol Biol Educ. 2024 Sep 4;53(1):15–20. doi: 10.1002/bmb.21860 (PMC11752409; doi:10.1002/bmb.21860)
Supplement: Supplementary file 1 — Data S1. Syllabus and intended learning outcomes for “Biochemistry: protein structure and function course” at Umeå University in Sweden. [file BMB-53-15-s002.docx]

# Biochemistry: protein structure and function (5KE170)

**Course Code:** 5KE170

**Responsible Department:** Department of Chemistry

**Grading System:** Three-grade scale

**Level of Education:** Second cycle

## Contents

Proteins are biological macromolecules that are essential for life. They perform key functions, such as providing structure to cells and tissues, catalyzing metabolic reactions, transporting molecules, or transmitting signals. These functions are determined by their chemical properties and physical interactions.

This course covers the thermodynamic principles that lead to the structure, stability and interactions of proteins, and the theoretical basis of methodologies to study protein structure and function. The course provides hands-on experience with designing and performing experiments to investigate biophysical properties of proteins, and discussion on how these affect function. The course also covers computational tools to visualize the structure of proteins.

## Expected Learning Outcomes

At the end of the course, students should be able to:

- Identify different motifs and domains in protein structures and how these relate to function.
- Understand and use the thermodynamic principles that determine the structure and folding of proteins, how and these impact function.
- Understand and describe the methodologies to determine protein structure, and suggest which one is more adequate to study a particular protein.
- Use the principles of molecular recognition to understand interactions with proteins.
- Relate the theoretical framework of catalysis to its application to study enzyme function.
- Use computational tools to visualize protein structure and to predict their function.
- Design and perform experiments to study which aspects of the structure are important for the stability, structure, and function of proteins.
- Critically discuss the results of experiments designed to study protein stability, structure, and function.

## Form of instruction

The course will take the form of lectures, group exercises, demonstrations, and laboratory experiments. Participation in the laboratory moments is mandatory.

## Examination

The course will be examined through a written exam on the theoretical part, and written report and its verbal presentation and discussion on the laboratory part. The written exam is graded with Fail (U), Pass (G) or Pass with distinction (VG), and the laboratory part is graded with Fail (U) or Pass (G). For the whole course, the grades Fail (U), Pass (G) or Pass with distinction (VG) are awarded. To pass the whole course, all examinations and obligatory elements must have been passed. The grade constitutes an overall assessment of the results of the different parts of the examination and is not allocated until all mandatory elements are complete.

Those who pass an examination may not take the same examination again with the aim of achieving a higher grade.

A student who has failed a course or part of a course twice is entitled to request appointment of another examiner, unless there are special reasons against it (HF Chap. 6 § 22). Requests for a new examiner should be made to the Head of the Department of Chemistry.

## Literature

Kuriyan, John.; Konforti, Boyana.; Wemmer, David.

The molecules of life: physical and chemical principles

ISBN:978-0-8153-4188-8
