# Supplementary material for: Teaching protein structure and function through molecular visualization
Source: Biochem Mol Biol Educ. 2024 Sep 4;53(1):15–20. doi: 10.1002/bmb.21860 (PMC11752409; doi:10.1002/bmb.21860)
Supplement: Supplementary file 2 — Data S2. Tutorials for the molecular graphics sessions. [file BMB-53-15-s001.docx]

Molecular graphics


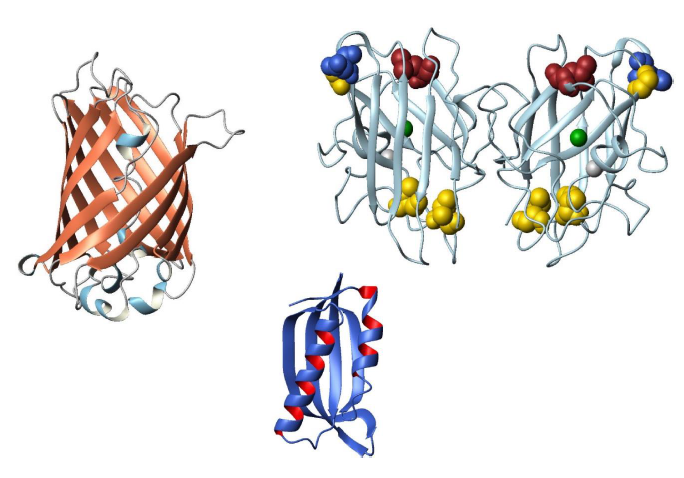


Biochemistry: Protein Structure and Function (5KE170)

Welcome to the molecular graphics section in the *Biochemistry: Protein Structure and Function (5KE170)* course. These sessions are meant to help you look at protein structures, and identify common structural motifs and how the structure relates to function.

The sessions are mandatory for all students taking the course. It is helpful if you come to the sessions prepared. Read the instructions and when you will be asked to identify certain motifs, it is important that you know what they are. Revisit the slides from the lectures, or google the different motifs and read about their physicochemical characteristics.

During the sessions you will be responsible for guiding yourself. The teachers will be there just to answer any questions that you have. They will not be checking if you have done all the exercises. The exercises are designed to make you familiar with different structures, interactions, physicochemical characteristics of amino acids, and thus helping you to answer questions on the exam. It is suggested that if you do not have time to complete all the exercises that you continue at home. We will be using a free software termed ChimeraX that you should be able to also install on your personal computer.

We hope you enjoy these sessions!

# Session 1: Introduction to molecular graphics

In these sessions, you will be using the program Chimera X to visualize protein structures. We will look both at structures determined experimentally by for example X-ray crystallography or cryo-EM, or predicted structures by for example AlphaFold. AlphaFold is a revolutionary algorithm introduced in 2021 that uses AI to predict protein structures with high accuracy. You will learn more about it during one of the lectures.

Today, you will familiarize yourself with the program, by learning to open structures, changing the appearance of the molecule, selecting and highlighting different parts of the molecule, measuring distances and aligning and overlaying structures.

In many cases, if you do not know how to do something, you can try simply googling it, someone else has probably gone through the same problem you are trying to solve.

Also helpful, if you find yourself working with ChimeraX a lot in the future, is to follow the tutorials offered by the developers of the software ([https://www.cgl.ucsf.edu/chimerax/ tutorials.html](https://www.cgl.ucsf.edu/chimerax/%20tutorials.html)). In these sessions, we will just be scratching the surface of what you can do with this piece of software.

## Task 1: Getting familiar with visualizing structures

1. Open a PDB structure, for example the protein of unknown function YbaA (2OKQ). To do this either download the PDB file from PDB (<https://www.rcsb.org/>) or type in the command line (at the bottom of the ChimeraX window) “open 2OKQ”.
   1. Notice that every time you perform an action it gets logged on the right side. You can perform the exact same action by typing that into the command line. This allows you to write “scripts” in a text file and reproduce a figure – for example if you want to make a figure for a report.
2. Familiarize yourself with what the mouse buttons do (rotate, zoom, translate). Explore the “right mouse” tab on top of the window – here you can select what a right mouse click does.
3. Explore the “molecule display” tab.
   1. Change between the different display modes (atoms, ribbons, surfaces).
   2. Explore coloring the charge (electrostatic) and hydrophobicity of the protein.
   3. Note that you can select only a part of structure and change the visualization just for that region (in the “right click” tab you can use a right click to select amino acids; either draw a box around a region or hold the shift key and select and deselect different regions; you can deselect everything by right clicking outside the structure).
   4. Select two of the beta sheets next to each other and explore the hydrogen bonding. To select the beta sheets, go in “Select>Structure>Secondary Structure> Strand”. To explore the hydrogen bonding, select to view atoms and hide the ribbons so that you can see the protein backbone. Perhaps hide the side chains (you can select only the side chain by using the menu “Select>Structure>Side chain only”, then see if you can find how to hide it). To view hydrogen bonds, select the two beta sheets and go to the tab “Molecule display” and select H bonds in analysis.
   5. Look also at hydrogen bonding in an alpha helix. Notice how polarized they are.
   6. When the structure of this protein was determined, it was found to be a dimer. You can see this by coloring by chain in the “Molecular display” tab. To visualize the surfaces and hydrogen bonds better you can try to hide one of the chains.
4. Try highlighting certain parts of the structure. This would be a good way to make a figure for a report when you want to highlight a certain feature.
   1. For example try coloring all the helices in one color and the sheets in another (hint: look at the “Select>Structure>Secondary structure” menu and “Action>Color” menu).
   2. Highlight also just a single helix by making the rest of the structure transparent (hint: select the helix you want to highlight (you can select a single amino acid of the helix and the Select>Broaden or simply press the arrow up), invert the selection, and by choosing a custom color set the alpha to a lower value (alpha is a value of transparency: 0 being fully transparent and 255 being fully opaque).

To set a custom color and change transparency go to “Action>Color>Custom...”

1. Try selecting parts of the sequence
   1. Open “Tools>Sequence>Sequence Viewer”
   2. You can click or click and drag to select different parts of the structure. You can then give them a color.
2. Explore the sequence pane (you can get it from the “Molecule display” tab). You can click on different parts of the sequence and highlight them in the structure. What does the color-coding mean? Google it if you are not sure.
3. Look at the Debye–Waller factor (you can color your structure by this “b-factor”). This factor is a measure of how rigid the structure is (blue rigid and red more flexible). Which parts are more flexible?
4. Go to PDB and read about the molecule of the month and explore some of the structures highlighted in the same way as you have done above, and explore the structure in a similar manner.

## Task 2: Calculate distances between atoms

1. ChimeraX allows you to calculate the distances between different atoms (it does not work if you select the whole amino acid) or between markers.
   1. Place a few markers around: in the tab “Markers” you can select “point” and right click to place a marker. Rotate the structure around to see if you placed the marker where you wanted it; you can use the move mode to move around the marker to the right position.
   2. If you place two markers you can measure the distance between them. Try “Tools>Structure Analysis>Distance”. In the new menu on the right side (if you have selected two markers) and click create you will be able to see the distance. Make sure you select both Markers (Go to the “right mouse” tab, click on “select” then select the 2 markers using right click + Shift key).
   3. If you place three markers you can calculate the angle that they make in the menu “Tools>Structure Analysis>Angles”.
2. Calculate the distance of one turn of an alpha helix. Do you get a similar value as stated in the lecture?
3. The structure of YbaA includes two alpha helices at an angle, calculate the angle formed.

## Task 3: Overlaying structures (**you might want to open a new ChimeraX session to do these exercises**)

1. ChimeraX allows you overlay structures and find out how different they are by calculating the root mean squared deviation, RMSD (google it or ask your favorite large language model to understand how it is calculated and what it means). We can compare how good an AlphaFold prediction is by overlaying it on top of a determined structure. Let’s compare the determine structure of YbaA (2OKQ) with its predicted AlphaFold structure.
   1. Open the AlphaFold structure for YbaA of *E. coli*. AlphaFold structures can be found based on their UniprotID (for example, in this case P0AAQ6 <https://www.uniprot.org/uniprotkb/P0AAQ6/entry>). Either download the AlphaFold structure from uniprot, or type “alphafold fetch P0AAQ6” in the command line. What does the color coding represent (use google)?
   2. Open the 2OKQ structure in the same session. You can see that there are 2 chains (and select them) in the log. You can also hide or delete chain B after opening it; to delete select chain you want to delete and use “Action>Atoms/Bonds>Delete”. Note that in the models you can also hide a whole structure if you want to pay attention to only one structure at a time.
   3. To overlay the structure, use the “Tools>Structure Analysis>Matchmaker” command. Select the experimentally determined structure (2OKQ) as your reference and the AlphaFold model (P0AAQ6) the structure to match. Click on “OK”. Appreciate how good the overlap is visually. On the right side you can see the RMSD (generally you want to consider all pairs, not just the pruned atoms), a value below 1 Å means that on average the atoms are within 1 Å of the reference, which is considered an excellent result!
   4. You can also look at the sequence alignment. When you are in the Matchmaker menu, you go to the tab sequence alignment and select “Show pairwise sequence alignment”. You will learn more about sequence alignment and the algorithms used during the bioinformatics lecture. You can also do better sequence alignments in UniProt.
   5. Are there any parts of the structure that do not align so well? Are the sequences really the same? Why do you think there is some extra sequence on the experimentally determined protein?

## Task 4: Practice exercises

1. Three different riboflavin kinases are available in the PDB: Human (1P4M); Schizosaccharomyces pombe (1N07); Methanocaldococcus jannaschii (2VBV)
   1. Two of these are structurally similar, which?
   2. Make a superposition of the structures and see which pair give the lowest RMSD.
   3. Hints: To make it easier to visualize hide chains; it might be easier to also hide extra chains and ligands; it might also be easier to do it in pairs.
2. Look at these two structures from heme oxygenase from *Corynebacterium diphtheria* (1IW1, 1IW0).
   1. Navigate to PDB to see what is different between them (the metadata associated with each structure is always an important starting point to know what you are looking at).
   2. Align them with matchmaker and see what looks different (hint: keep only chain A and delete the other ones)
   3. It might be easier to see the differences by animating the structure transitioning from one to the other. Google or ask your favorite large language model how you might be able to do that (hint: the command is called “morph” and it might be good to turn “wrap true” so that the structure bounces back to the initial position).

# Session 2: Motifs in protein structures

In this session, you will be exploring different motifs in proteins, particularly focusing on basic motifs with alpha helices and beta-sheets. You will be given quite broad exercises. The idea is that you familiarize yourself with the major motifs and their physicochemical characteristics (size, hydrophobicity of different parts of the structure, charge, amino acid composition, etc). For some motifs you will be hinted to look at a particular aspect, but these hints should only be the starting point. You should use all the different the ways you learned in the previous session to explore these structures. Read more about the motifs (in your textbook or online)! In the final written exam, there are usually a few questions about motifs, and these are not covered during the lectures, but only here.

When you look at a PDB structure, it is always good to go to PDB and see what structure you are looking at, how it was acquired, which organism does the sequence come from, how it was expressed, does it have any mutations, are there any publications linked to it?

## Task 1: Secondary structure

1. Explore the structure of human carbonic anhydrase II (2CBA).
   1. Identify parallel and anti-parallel beta-sheets.
   2. Pay attention to how close in the linear sequence both types of sheets are.
   3. Look at the hydrogen bonding of the backbone for both types of beta-sheets.
   4. The beta-sheets are not just flat, but have a certain turn, pay attention to it. Is it right or left turn?
   5. Look also at the alpha helices and the hydrogen bonding. Pay also attention to how the side chains are oriented and if there are potential weak interactions between the side chains (electrostatic, hydrogen bonds, van der Waals).

Hint: you can color specific amino acids (for example: positively charged) by writing in the command line “select : Arg, Lys” (this will select the amino acids) and by going to “Action>Color”. This will allow you to select all the same type of amino acids in one go and give them all the same color.

- 1. Look at which amino acids in the helices are facing the inward part of the structure and which ones are facing the solvent. Do you notice any physicochemical characteristics of each type of amino acid?

Hint: Color and show the side chains of the polar/ non-polar amino acids on the helices. (You can make more transparent the rest of the structure)

1. Look at the structure of adenylate kinase (1AKE).
   1. You will notice that the long alpha helix on the side of the protein appears to be separated in two. Investigate why that might be the case (hint: it probably helps to visualize the amino acids and their side chains for that particular region; try to hide the rest of the structure to be able to focus just on that region).
2. Look at the structure of the human growth hormone (1HGU).
   1. Pay attention to the 4-helix bundles. Some amino acids face the exterior of the protein while others face the interior. Which type of amino acids is more towards the outside and which one more towards the inside?

To do this, you can try to show the hydrophobic surfaces, but it is difficult to peer into the inside of the protein. One possibility is to color the different types of amino acids (hydrophobic, polar, negatively and positively charged) with different colors.

- 1. Look at the side chains of the alpha helices and see how they are packed and what kind of interactions form between the different residues. It might be easier to look at one helix at a time, so that the structure is not too crowded.

## Task 2: Motifs

1. Look at the structure of Troponin-C (1NCX)
   1. Pay attention to the EF-hand motif.
   2. Look at the binding of calcium (there are no calcium ions in the structure but two cadmium).
   3. Look at which atoms are involved in the coordination of the divalent cations.
   4. Which residue numbers in the sequence match the conserved sequence? (Remember there are 2 calcium binding motifs)

Hint: The bigger the amino acid in the motif the more conserved it is at that position.


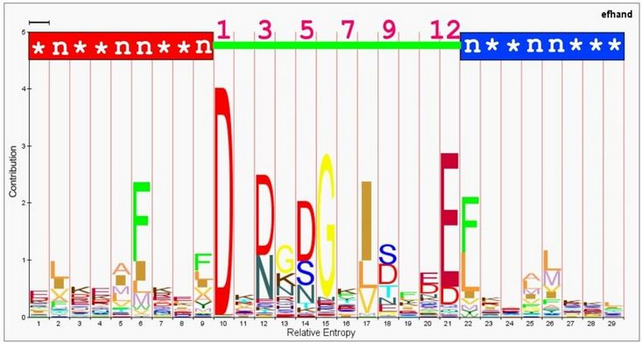


1. Look at the structure of Bovine pancreatic trypsin inhibitor (1AAL).
   1. Pay attention to the hairpin-motif (hint: there are two similar structures but only one is a hairpin-motif).
   2. Which amino acids form this hairpin motif?
2. Look at the structure of *Staphylococcus* nuclease (1A2T).
   1. Pay attention to the Greek key motif.
   2. Follow the chain and compare it to the schematic/flat representation of the motif that you see in the textbook.
3. Look at the structure of Triose phosphate isomerase (1YPI).
   1. Pay attention to the β-α-β motif.
   2. In the UniProt page related to this protein (under function, <https://www.uniprot.org/uniprotkb/P00942/entry>) you can find the residues that form the active site. Look at these residues and their side chains.

Hint: To select specific residues you can type in the command line “select :” and the number of the residues. (e.g., to select A23 and H60, type in “select : 23, 60”)

1. Look at the structure of flavodoxin (1RCF).
   1. Pay attention to the open twisted alpha-beta sheet, which is a common way to attain parallel beta-sheets.

# Session 3: Larger structures

In this session, you will be looking at how motifs organize themselves in larger structures. You should pay attention to the characteristics of amino acids that form each motif. Which type of amino acid is exposed to the solvent and which one is buried? If you see amino acids with charge in a hydrophobic environment, do you see other amino acids that could compensate for that charge? How are hydrogen bonds important to stabilize the structures?

## Task 1: Barrels

1. Look at the structure of Superoxide dismutase (1SXC).
   1. Pay attention to the beta-barrel.
   2. As usual, look at the amino acid composition on the inside and outside of the protein.
   3. Look also at hydrogen bonding and other types of interactions.
2. Revisit the structure of Triose phosphate isomerase (1YPI) from the previous session.
   1. This time pay attention to the TIM barrel.
   2. Look again at which amino acids face the interior of the protein (the beta-sheets and the inside of the helices) compared to the ones on the outside of the alpha helices.
3. Look at the structure of the Retinol binding protein (1BRP).
   1. Pay attention to the up and down barrel.
4. Look at the structure of neuraminidase (1NNC).
   1. Pay attention to the super barrel.
   2. Notice that the structure of anti-parallel beta-sheets repeats itself 6 times.
5. Look at the structure of Concanavalin A (3CNA).
   1. Pay attention to the jelly roll motif.

## Task 2: Membrane proteins

1. Look at the structure of the sucrose-specific porin (1A0S​).
   1. Look at the amino acid characteristics of this protein. Remember that this is a membrane protein.
   2. Pay attention to which amino acids form the beta-sheets. You can focus on a single beta sheet and look how the side chains are oriented. Do you see any patterns on the side chains depending on where they are pointing?
   3. Think about what is the function of this protein (and look it up to see if you were right).
2. Look at the structure of bacteriorhodopsin (1C8S).
   1. Do a similar analysis as for the porin.

## Task 3: Repeated structures

1. Look at the structure of Pectate lyase (1IDK).
   1. Pay attention to the beta-helix structure.
2. Look at the structure of the ribonuclease inhibitor (2BNH).
   1. Pay attention to the horseshoe structure with repeated beta-loop-alpha motifs.
   2. Look at the sequence of the beta-sheets. Anything that is common between most of them? Hint: there is another name for the motif that constitutes the horseshoe structure that is related to the amino acid composition (google or ask your favorite large language model it if you cannot identify it yourself).
   3. In the structure 1DFJ, you can see the ribonuclease inhibitor in complex with ribonuclease A. Does that offer any clues to how the inhibitor works?

## Task 4: Catalytic activity

1. Look at the structure of trypsin (1MCT).
   1. This is a serine protease, which has a known mechanism (look it up).
   2. In this structure, the catalytic triad is formed by Ser 195, His 57, Asp 102. Look at their side chains and look at the mechanism of catalysis (in your textbook or by googling it) to understand how a serine protease works.

# Session 4: DNA recognition

In this session, you will look at the structure of DNA and how proteins bind DNA and recognize different sequences. As usual, think about interactions. DNA has a negatively charged backbone. How do you expect proteins to bind DNA?

## Task 1: DNA structures

1. Look at the structure of B-DNA (127D).
   1. Identify the major and minor groove.
   2. Contrast this structure with Z-DNA (145D). If you do not remember the different types of DNA look them up.

## Task 2: DNA recognition

1. Look at the structure of the Cro repressor (6ON0).
   1. Look at the electrostatic interactions that allow it to interact with DNA.
   2. Look at the helix that interacts with DNA.
   3. Look at the side chains of this helix and the possible interactions with DNA.
2. Look at the structure of the 434 repressor (1PER).
   1. Do a similar analysis as for the Cro repressor.
   2. Notice that they recognize different nucleotide sequences, so there must be some specificity in the interactions that the side chains of the amino acids form with the bases of DNA.
3. Look at the structure of the TATA box binding protein bound to DNA (1RM1).
   1. Look at how much the DNA is bent.
   2. Pay attention to the interactions that are formed between the protein and DNA.
4. Look at the structure of p53 (1TUP).
   1. Look at the interactions with DNA.
   2. Pay attention to the structure of DNA.
5. Look at the structure of ZIF268 zinc finger (1AAY).
   1. As above look at how it interacts with DNA.
   2. What kind of nucleotide sequences do zinc fingers usually bind?
   3. Pay attention to how zinc is coordinated in this protein.
6. Look at the structure of a leucine zipper (2ZTA) with alpha helices forming a coiled-coil domain.
   1. Look at the amino acids (and side chains) that form the interactions in this dimer and how they relate to the name of the protein.

# Session 5: Getting hints on protein function using computational tools

In this session, you will look at a protein of uncharacterized function and using multiple computational tools to try to predict its function. Many of these tools were only recently developed and made possible by large advances in artificial intelligence.

Your task is to use the tools below, or others that you have heard of or find by googling or asking some large language model, and try to get the first hints of function. You will be working at the frontier of knowledge, so you are not expected to end this session with the knowledge of what the protein is doing. This could be the starting point to start studying the function of these proteins based on knowledge of how proteins with a similar structure/sequence function. For example, if you find out that your protein looks like a transcriptional regulator you could develop biochemical assays to identify which DNA sequences it prefers to bind. This could then open the door for you to look for those sequences in the genome and try to identify which genes are regulated by your protein.

Here are UniProt entries for proteins that are currently uncharacterized (feel free to find some other uncharacterized protein):

- A6KXT0
- P0AAY6
- A6KXI0
- Q8RE61
- Q8RE88
- A0A174A107
- R6AX79
- R6AY32
- Q189Q8
- Q183W3

## UniProt

UniProt is a database that collects information about virtually all the proteins that are known. This database links to many other useful databases, and for proteins that are characterized there can be a lot of curated information in there. Here is an example of a well characterized protein (<https://www.uniprot.org/uniprotkb/P69441/entry>). See the amount of information that you can find and compare it to your uncharacterized protein.

Look at what organism your protein comes from. See if you can learn something about that organism.

## InterPro

In UniProt, you can sometimes get hints on the function by looking at the domains that are present in the protein. This will take you to the InterPro database which classifies proteins into different families based on their structure.

## AlphaFold and ColabFold

From UniProt, you can also download the AlphaFold predicted structure and visualize it in ChimeraX. All the proteins in UniProt have a predicted AlphaFold structure, but if in the future you are given a sequence and want to predict its structure you can use ColabFold (<https://colab.research.google.com/github/sokrypton/ColabFold/blob/main/AlphaFold2.ipynb>). You can just paste the amino acid sequence of your protein and click the “play” buttons on the left side. For some proteins this can take a few hours. Since you already have a predicted structure, you do not need to do this today.

Looking at the predicted structure are there any motifs that you can recognize from the past sessions? Any particular helices/sheets that could hint if the protein is embedded or anchored to the membrane based on the amino acid composition?

## Sequence similarity (BLAST)

At the bottom of the UniProt page for your protein you can explore other proteins with similar sequences to your protein (at 100%, 90% and 50% cutoffs). This was performed with a BLAST search that you learned about in the bioinformatics session. See if you find proteins in there with similar sequences and with a characterized function (or with a name that suggests that they have a known function). From there go into their UniProt page and see if there is more information. You can also download their structures (or predicted AlphaFold structures) and use Matchmaker in ChimeraX to see if they have a similar structure to your protein.

## Structure similarity (Foldseek and Dali)

It is more likely that proteins of similar function retain their structure than their sequence. Thus, comparing structures to those of proteins can be more powerful than doing it based on sequence. One tool that can do this extremely fast is Foldseek ([https://search.foldseek.com/ search](https://search.foldseek.com/%20search)). You can upload your AlphaFold PDB and search it there (there is also a direct link from the UniProt page of your protein under the structure; you still need to click search after you are directed to Foldseek). This will compare the structure to all the AlphaFold structures in UniProt as well as structures that are in the PDB. You are more likely to find proteins with characterized function in the PDB. When you look at your results, they are sorted from the best to the worst match in each database. If you click under “alignment” for one of the results you can see a sort of “Matchmaker” between your protein and the protein that was matched. Be careful, because you might only have structure similarity to a very small portion of your protein, or it might not be the part that is important for function! If the alignment looks good, you can download the PDB of the protein that was previously characterized and do the Matchmaker in ChimeraX to really have a good look at how the two structures align.

An older algorithm called Dali (<http://ekhidna2.biocenter.helsinki.fi/dali/>) was used to compare protein structures to entries in the PDB. This is a much slower algorithm, so it does not scale with the size of the AlphaFold database, which currently has >200 million predicted structures (vs around 200 thousand structures in the PDB).

## Prediction of function using AI (DeepFRI)

DeepFRI (<https://beta.deepfri.flatironinstitute.org/>) is a tool that has been trained to identify the function of motifs based on the structure and/or sequence of proteins. If you upload a structure, it will give you predictions of gene ontologies for different parts of your structure and sequence. These can sometimes give hints of function.
